# Unveiling the Genetic Blueprint of a Desert Scorpion: A Chromosome-level Genome of *Hadrurus arizonensis* Provides the First Reference for Parvorder Iurida

**DOI:** 10.1093/gbe/evae097

**Published:** 2024-05-03

**Authors:** Meridia Jane Bryant, Asher M Coello, A M Glendening, Samuel A Hilliman, Carolina Fernanda Jara, Samuel S Pring, Aviel Rodríguez Rivera, Jennifer Santiago Membreño, Lisa Nigro, Nicole Pauloski, Matthew R Graham, Teisha King, Elizabeth L Jockusch, Rachel J O’Neill, Jill L Wegrzyn, Carlos E Santibáñez-López, Cynthia N Webster

**Affiliations:** Department of Ecology and Evolutionary Biology, University of Connecticut, Storrs, CT, USA; Department of Ecology and Evolutionary Biology, University of Connecticut, Storrs, CT, USA; Department of Ecology and Evolutionary Biology, University of Connecticut, Storrs, CT, USA; Department of Ecology and Evolutionary Biology, University of Connecticut, Storrs, CT, USA; Department of Ecology and Evolutionary Biology, University of Connecticut, Storrs, CT, USA; Department of Ecology and Evolutionary Biology, University of Connecticut, Storrs, CT, USA; Department of Molecular and Cell Biology, University of Connecticut, Storrs, CT, USA; Department of Physiology and Neurobiology, University of Connecticut, Storrs, CT, USA; Institute for Systems Genomics, University of Connecticut, Storrs, CT, USA; Department of Molecular and Cell Biology, University of Connecticut, Storrs, CT, USA; Institute for Systems Genomics, University of Connecticut, Storrs, CT, USA; Department of Biology, Eastern Connecticut State University, Willimantic, CT, USA; Department of Ecology and Evolutionary Biology, University of Connecticut, Storrs, CT, USA; Department of Ecology and Evolutionary Biology, University of Connecticut, Storrs, CT, USA; Department of Molecular and Cell Biology, University of Connecticut, Storrs, CT, USA; Institute for Systems Genomics, University of Connecticut, Storrs, CT, USA; Department of Ecology and Evolutionary Biology, University of Connecticut, Storrs, CT, USA; Institute for Systems Genomics, University of Connecticut, Storrs, CT, USA; Department of Biology, Western Connecticut State University, Danbury, CT, USA; Department of Ecology and Evolutionary Biology, University of Connecticut, Storrs, CT, USA; Institute for Systems Genomics, University of Connecticut, Storrs, CT, USA

**Keywords:** scorpion, arachnid, Hadruridae, reference genome, pore-c, nanopore

## Abstract

Over 400 million years old, scorpions represent an ancient group of arachnids and one of the first animals to adapt to life on land. Presently, the lack of available genomes within scorpions hinders research on their evolution. This study leverages ultralong nanopore sequencing and Pore-C to generate the first chromosome-level assembly and annotation for the desert hairy scorpion, *Hadrurus arizonensis.* The assembled genome is 2.23 Gb in size with an N50 of 280 Mb. Pore-C scaffolding reoriented 99.6% of bases into nine chromosomes and BUSCO identified 998 (98.6%) complete arthropod single copy orthologs. Repetitive elements represent 54.69% of the assembled bases, including 872,874 (29.39%) LINE elements. A total of 18,996 protein-coding genes and 75,256 transcripts were predicted, and extracted protein sequences yielded a BUSCO score of 97.2%. This is the first genome assembled and annotated within the family Hadruridae, representing a crucial resource for closing gaps in genomic knowledge of scorpions, resolving arachnid phylogeny, and advancing studies in comparative and functional genomics.

**Significance**

Genomic resources for the study of arachnids are limited. To date, only four scorpion genomes have been published; none of these are chromosome-level assemblies, and all four belong to a single family, Buthidae. In this study, we assembled the first chromosome-level, annotated genome for a nonbuthid species (*Hadrurus arizonensis*). This high-quality reference will provide a critical resource for comparative and functional genomics and contribute to the understanding of arachnid evolution.

## Introduction

Arachnids emerged on land over 400 million years ago (Coddington and Colwell 2001) and are a diverse taxonomic group containing over 100,000 known species (Proctor et al. 2015). They inhabit a range of habitats, including terrestrial and aquatic environments (Kuntner 2022). Despite this diversity, genomic research within Arachnida has primarily focused on Araneae (spiders) (Sanggaard et al. 2014) and Parasitiformes (ticks) (Pagel Van Zee et al. 2007).

The order Scorpiones comprises 23 families and over 2,800 species in two main parvorders: Buthida and Iurida (Santibáñez-López et al. 2022, 2023). Most are nocturnal, solitary predators and the majority of species fluoresce under UV light. Being Arachnopulmonates, they possess book lungs for respiration; however, unlike other arachnids, their body is uniquely segmented into a prosoma, mesosoma, and metasoma (Howard et al. 2019). They have a pair of chelate pedipalps for defense and prey acquisition (prosoma), ventral sensory organs called pectines (mesosoma), and a segmented tail that ends in a telson and stinger (metasoma) to deliver venom. Scorpion venom is often the subject of research for human health and biotechnological applications (Kerkis et al. 2017). Despite this, significant gaps persist in regards to the diversity and evolution of scorpions (Kuntner 2022).

The higher-level relationships among scorpions have been controversial, especially with regard to monophyly and trait evolution (Lozano-Fernandez et al. 2019; Santibáñez-López et al. 2020; Ballesteros et al. 2022). The contribution of more high-quality genomes from under-represented families, apart from the four buthid genomes currently available, can improve our understanding of relationships among scorpions and the evolution of unique traits (Long et al. 2003; Rittschof and Robinson 2016).

**Fig. 1.**
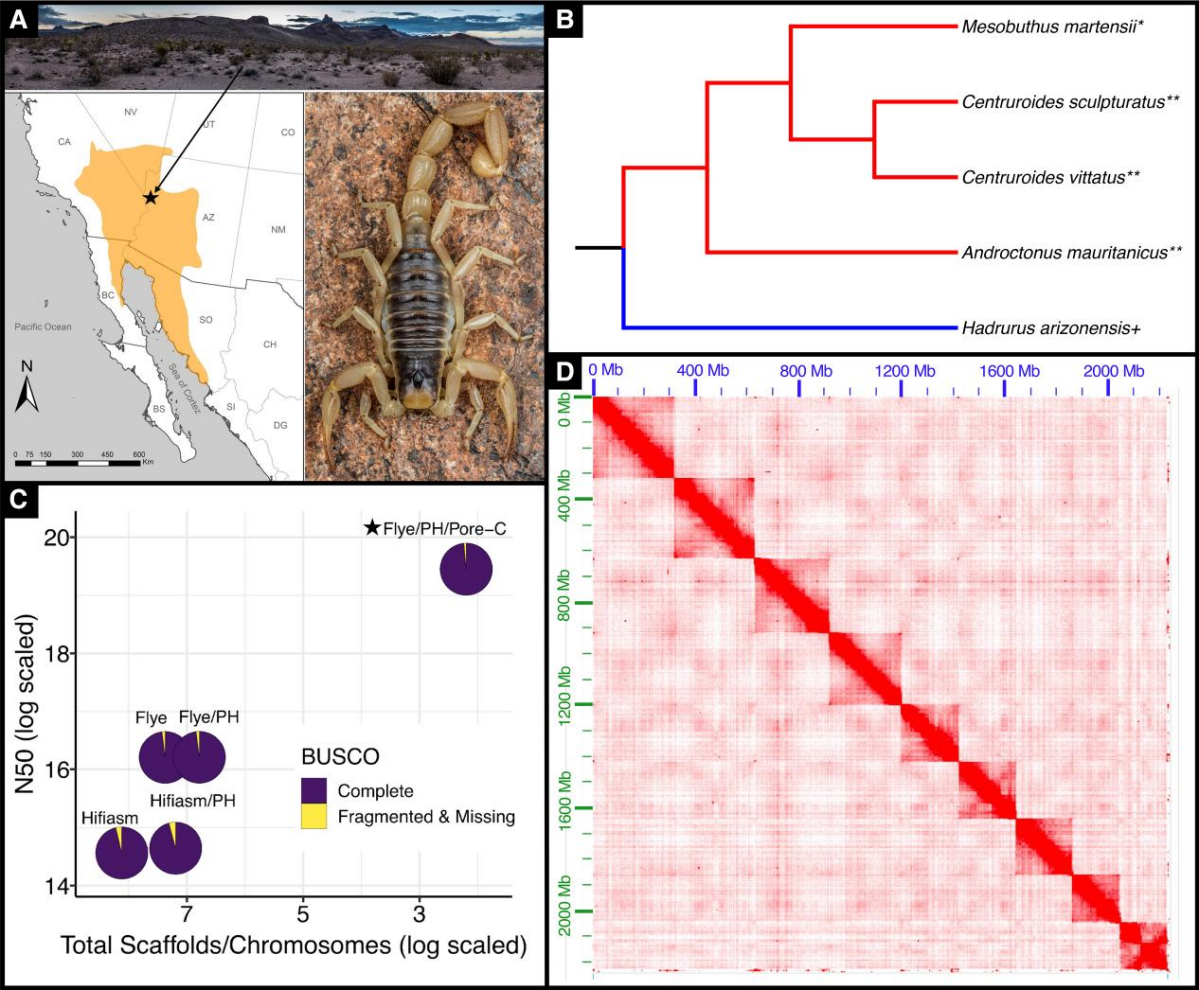
A) Range map of *H. arizonensis* with a point (star) denoting the approximate sampling location. The arrow extends to a photo of this region (near Oatman, Arizona, USA). The image on the right is a photo of *H. arizonensis* [both images are courtesy of Brent E. Hendrixson]. B) Phylogeny of scorpion species with genomes currently available on NCBI, informed by previous molecular phylogenetic analyses (Lozano-Fernandez et al. 2019; Santibáñez-López et al. 2023). Symbols appended to the species name indicate assembly status: “*” Scaffold level, “**” Contig level, “+” Complete chromosome-scale assembly. The lighter shade (red) segments indicate parvorder Buthida while the darker shade (blue) segments indicate parvorder Iurida. C) Contiguity (N50 and total number of scaffolds/contigs) and completeness (BUSCO) of genome assembly approaches for *H. arizonensis*. These include Flye and Hifiasm, with and without purge haplotigs (PH). The final chromosome-scale assembly is denoted with a star. Each assembly is represented by a pie chart to visualize BUSCO completeness. The darker shade (dark purple) indicates complete BUSCOs while the lighter shade (yellow) indicates missing and/or fragmented BUSCOs. D) Chromatin contact map generated from Pore-C data shows the nine chromosomes (2*n* = 18) that represent 99.56% of the assembled *H. arizonensis* genome.

This study presents the first chromosome-scale genome assembly for the desert hairy scorpion, *Hadrurus arizonensis*. This species is an iconic inhabitant of the Mojave and Sonoran deserts of North America, and one of the largest scorpions found on the continent (Fig. 1A). It also provides the first genome assembly for any species of the parvorder Iurida (Fig. 1B). The sequenced genome of *H. arizonensis*, known to be significantly impacted by historical climate shifts (Graham et al. 2013), also offers a valuable foundation for future studies of adaptation in extreme environments.

## Results and Discussion

### Sequencing

Nanopore sequencing generated 13,570,836 high-quality long reads, of which 92.8% were sheared (read N50: 14,664 bp) and 7.2% were ultralong (read N50: 52,738 bp). Following contaminant screening, 63,023 reads (0.46%) were removed (supplementary table S1, Supplementary Material online). The k-mer based genome size estimation was 2.31 Gb from 109.96 Gb of read data (47.6×), double the 1.1 Gb GoaT estimation (Challis et al. 2023) (supplementary table S1, Supplementary Material online).

### Genome Assembly

Flye and Hifiasm, with and without Purge Haplotigs (PH), were used to assemble the first genome (Fig. 1C; supplementary table S1, Supplementary Material online). The assemblies ranged from 2.2 Gb to 2.6 Gb in length. Flye had an N50 of 10.9 Mb with 1.6 K contigs, and a BUSCO score of Complete (C): 98.1%, Duplicated (D): 3.7%, and Fragmented & Missing (F&M): 1.9%. Hifiasm had an N50 of 2.1 Mb with 3.4 K contigs, and a BUSCO score of C: 96.3%, D: 12.2%, and F&M: 3.7%. Hifiasm PH had an N50 of 2.3 Mb and 1.3 K contigs, with a BUSCO score of C: 95.8%, D: 6.2%, and F&M: 4.2%. Flye PH had the highest N50 of 10.97 Mb and the fewest contigs (885 in total) (supplementary table S1, Supplementary Material online). Flye PH had a BUSCO score of C: 98.1%, D: 3.7%, and F&M: 1.9%. Flye PH, with the greatest contiguity and completeness, was selected for scaffolding. Contigs less than 3 Kb were removed from Flye PH prior to scaffolding. The final filtered Flye PH assembly had an N50 of 10.9 Mb, 850 contigs, a BUSCO score of C: 98.2% [S: 94.6%, D: 3.6%], F&M: 1.8%, and a Merqury QV score of 41.27.

### Genome Scaffolding

The sequenced Pore-C reads yielded 86.9 Gb (∼37×) of data with a mean read quality of 19.8 and read length N50 of 4.3 Kb (supplementary table S2, Supplementary Material online). The wf-pore-c and YaHS pipeline reoriented all 850 contigs into 242 scaffolds (Fig. 1D). Of these, 9 represented 99.56% of the genome, while the remaining 233 accounted for 0.44%. The final chromosome-level assembly was 2.23 Gb in size and had an N50 of 280 Mb, BUSCO score of C: 98.6%, D: 3.4%, and F&M: 1.4%, and Merqury QV of 41.28. While karyotypes of *Hadrurus hirsutus* within Hadruridae suggest an approximate haploid number of 50 (Wilson 1931), scorpion haploid numbers vary significantly, ranging between 5 and 90 (Št'áhlavský et al. 2021).

### Repeat Annotation

Prior to structural annotation, RepeatMasker softmasked 1.22 Gb (54.69%) of the genome.

**Fig. 2.**
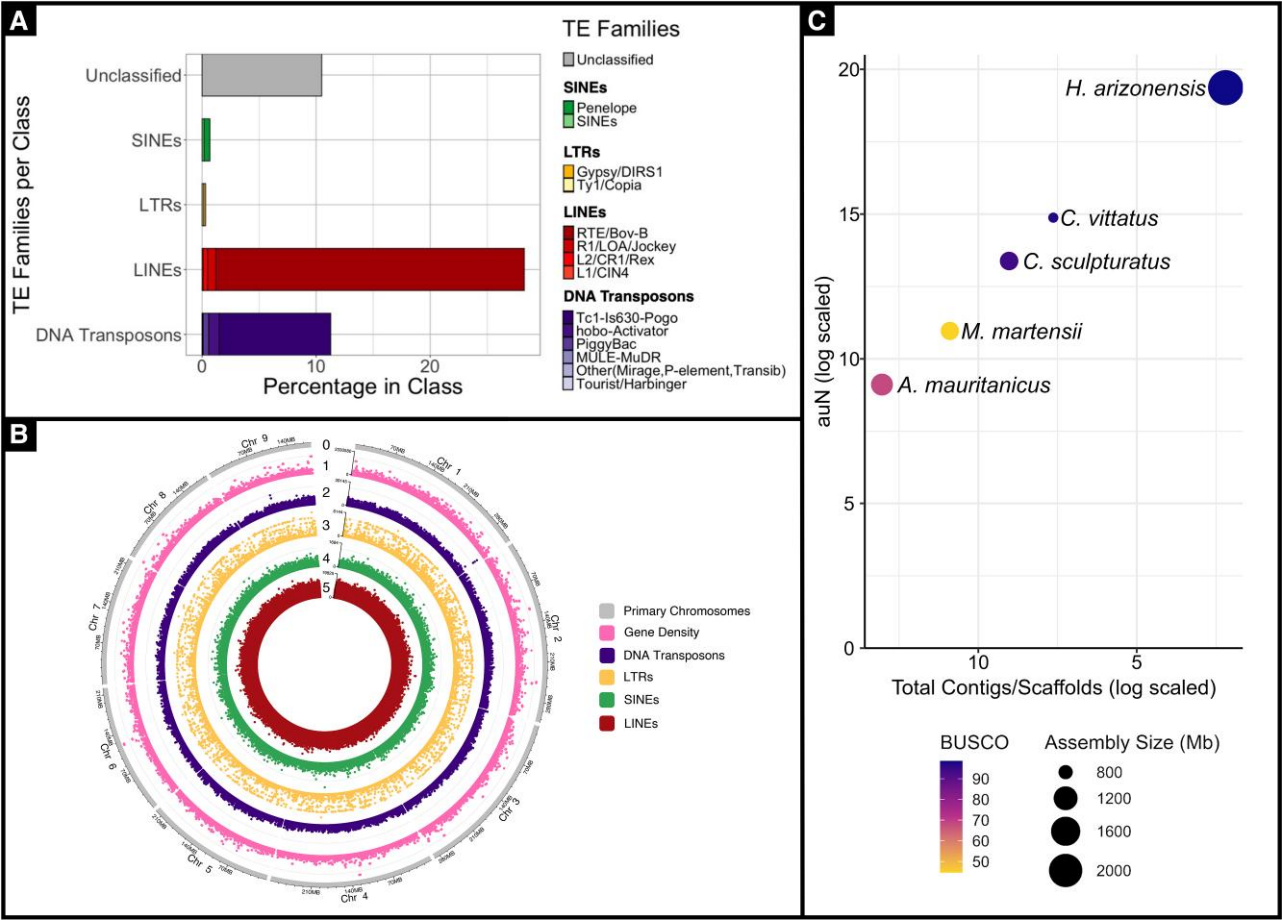
A) Percentage of TE families (by repeat class) in the *H. arizonensis* genome assembly. Color gradients were used to differentiate the families in each of the TE classes: Unclassified (gray), SINEs (green), LTRs (yellow), LINEs (red), and DNA transposons (purple) (supplementary table S3, Supplementary material online). B) ShinyCircos (v2.0) (Wang et al. 2023) representation of the chromosome-scale assembly with the following numbered tracks (10 Kb windows) from outside to inside: (0) primary chromosomes (gray), (1) gene density (pink), (2) DNA transposon density (purple), (3) LTR density (yellow), (4) SINE density (green), and (5) LINE density (red). C) Contiguity (auN and total scaffolds/contigs) and completeness (BUSCO) of the four scorpion genomes available in NCBI, in addition to *H. arizonensis*. The gradient (light to dark/yellow to dark purple) represents BUSCO completeness while the size of each point represents the assembly size (supplementary table S7, Supplementary Material online). The y-axis, auN, is a measure of contiguity provided by QUAST (Mikheenko et al. 2018).

Retrotransposons were the most abundant transposable element (TE) class in the *H. arizonensis* genome, comprising 29.86% of the repetitive elements identified (Fig. 2A). Long interspersed nuclear elements (LINEs) represented the majority of retrotransposons (29.39%), in stark contrast with short interspersed nuclear elements (SINEs) (0.16%) and long terminal repeat elements (LTRs) (0.31%) (supplementary table S3, Supplementary Material online; Fig. 2B). Interestingly, Bovine-B (RTE/Bov-B) was the most abundant LINE (27.07%). Bov-B elements have a widespread and patchy distribution in eukaryotes and phylogenetic analysis of these elements has identified potential horizontal transfer vectors in Arthropoda (Ivancevic et al. 2018). Approximately 13.57% of the genome’s repeat elements consist of DNA transposons, with Tc1-IS630-Pogo accounting for 9.77%. This family has been identified in several species, including *Drosophila*, plants, even vertebrates (Gao et al. 2020) and, in some instances, greatly expanded (Marburger et al. 2018).

### Protein-coding Genes

Six *H. arizonensis* RNA libraries exported from NCBI had an average mapping rate of 92.44% (supplementary table S4, Supplementary Material online). Combined, the filtered EASEL and GeneMark structural annotation yielded 31,841 genes and 90,343 transcripts. These numbers were reduced to 18,996 and 75,256, respectively, following PFAM (protein domain) filtration (Fig. 2B). With alternative transcripts present, the mono:multi exonic ratio was 0.135 and BUSCO completeness was 97.2% [S: 9.7%, D: 87.5%] . After taking the longest isoform, representing the unique gene space, BUSCO completeness was 91.6% [S: 87.6%, D: 4.0%] and the mono:multi exonic ratio remained mostly unchanged at 0.14 (supplementary table S5, Supplementary Material online). The reciprocal BLAST annotation rate of the longest isoform proteins against the RefSeq database was 59.21%. In comparison, the annotation rate for gene family assignment using the EggNOG v4 database was 74.33%. Combined, the final annotation rate for the extracted longest isoforms reached 75.94% (supplementary table S6, Supplementary Material online). While 18,996 genes may be an underestimation in comparison to the 24,591 predicted protein-coding genes in the *Centruroides sculpturatus* genome (GCF_000671375.1; Schwager et al. 2017), they are complete and in range with other Araneae (Thomas et al. 2020).

### Scorpion Genomes

Presently, only 12 of 237 arachnid genomes hosted at NCBI are chromosome-scale. Prior to assembling *H. arizonensis*, there were no chromosome-scale genome assemblies within the order Scorpiones. As such, this high-quality reference represents the first, with a completeness and contiguity that surpasses the scorpion assemblies available to date (Figs 1B and 2C).

## Materials and Methods

### Collection

One adult female *Hadrurus arizonensis* individual was collected near Oatman, AZ, USA on August 25th, 2022, and preserved in 100% ethanol (Fig. 1A). The legs and pedipalps were removed, rinsed in nuclease-free water, and frozen in liquid nitrogen. The tissue was ground in liquid nitrogen to a fine powder and stored at −80 °C.

### DNA Extraction

*Hadrurus arizonensis* DNA was extracted using the Monarch^Ⓡ^ high molecular weight (HMW) DNA Extraction Kit: Tissue Protocol (NEB, T3060). Changes made to the protocol include: adding 580 μL of HMW gDNA Tissue Lysis Buffer to approximately 30 mg of frozen ground tissue, lysis incubation was at 56 °C for 45 min at 700 rpm and the binding of the gDNA to beads was performed on a vertical rotating mixer at 8 rpm rather than 10 rpm. DNA concentration was measured using a Qubit fluorometer. The gDNA for the standard library was sheared using a Covaris^Ⓡ^ G-tube to approximately 25 Kb and small fragments were removed using PacBio's Short Read Eliminator XS kit. For the ultralong library, three separate Monarch^Ⓡ^ extractions were pooled and eluted in Oxford Nanopore's EEB buffer.

### Genomic Library Preparation

Oxford Nanopore Technologies^Ⓡ^ Ligation Sequencing Kit V14 (SQK-LSK114) was used to prepare the library on the extracted HMW DNA. DNA repair was performed at 20 °C for 20 min followed by 10 min at 65 °C to inactivate the enzymes. DNA was eluted at 37 °C. The final library was quantified using a Qubit fluorometer. The flow cell was loaded 3 times each with 10.5 fmol of library. For ultralong genomic library preparation, the Ultra-Long DNA Kit V14 (SQK-ULK114) was used on the pooled HMW DNA. Both PromethION flow cells ran for 72 h.

### Pore-C Library Preparation and Sequencing

A total of 150 mg of ground tissue was used for cell crosslinking. Crosslinked cells were resuspended in permeabilization solution and incubated on ice. The chromatin was denatured and the permeabilized cells were digested with restriction enzyme NlaIII. To reverse the crosslinking of the ligated chromatin, the sample suspension was incubated in a thermomixer with periodic rotation. Full details on the library preparation are available (supplementary file S1, Supplementary Material online). A total of 1.5 μg of purified DNA was used as input material for the SQK-LSK114 (Oxford Nanopore Technologies, UK) kit and protocol. DNA repair was performed at 20 °C for 20 min, followed by 65 °C for 10 min to inactivate the enzymes. The PromethION flow cell was run for 96 h.

### Genome Assembly and Scaffolding

Ultralong and sheared ONT raw reads were basecalled by Dorado (v7.0.8) and assessed with NanoPlot (v1.33.0) (De Coster and Rademakers 2023). Reads passing a quality threshold (Q > 10) underwent contaminant filtering using Centrifuge (v1.0.4-beta), with a minimum match length of 50 bp against NCBI's RefSeq bacteria, archaea, and fungi databases (Kim et al. 2016). Classified reads were removed, and NanoPlot was rerun. Genome size and coverage was estimated using kmerfreq (v4.0) and GCE (v1.0.2) at k-mer size 21 (Liu et al. 2013; Wang et al. 2020).

Two long-read de novo assembly tools, Flye (v2.8.1) and Hifiasm (v0.19.6-r595), were assessed (Kolmogorov et al. 2019; Cheng et al. 2021). Flye ran with coverage set to 60, while Hifiasm ran with default parameters. Assembly completeness, contiguity, and accuracy were estimated at each assembly stage with BUSCO (v5.4.5) using the arthropoda_odb10 lineage database, Merqury (v1.3), and QUAST (v5.2.0), respectively (Mikheenko et al. 2018; Rhie et al. 2020; Manni et al. 2021). Both assemblies were polished with Medaka (v1.9.1) using model r1041_e82_400bps_sup_g615 and minimap2 (v2.26) aligned raw reads, but neither moved forward due to decreased completeness and quality (*medaka* n.d.; Li 2018). To reduce duplication, Purge Haplotigs (v1.1.2) was run on the original assemblies (Roach et al. 2018). Low, medium, and high read-depth thresholds were set to 9, 26, and 195 in Flye, respectively, and 3, 32, and 195 in Hifiasm. The purged Flye assembly was selected for scaffolding due to superior BUSCO completeness, N50 (contiguity), and accuracy (Merqury QV).

A single Pore-C library was basecalled by Dorado (v7.1.4) and used to scaffold the Flye PH assembly. The wf-pore-c (v1.0.0) Nextflow workflow was employed to preprocess and align Pore-C raw reads to the draft genome with Fastcat (v0.14.1) and minimap2 (v2.26-r1175), respectively (*wf-pore-c*). The resulting alignments were converted into a 4DN-format pairs file with pairtools parse2 (v1.0.2) (Open2C et al. 2023). YaHS (v1.1) conducted multiple rounds of scaffolding using the pairs file format, genome, and “GATG” restriction enzyme flag. The resulting Pore-C alignment and APG file were run through juicer (v1.2) pre and juicer_tools (v1.9.9) for visualization with Juicebox (Durand, Robinson, et al. 2016; Durand, Shamim, et al. 2016; Zhou et al. 2023). The nine manually curated chromosomes were assessed with BUSCO, Merqury, and QUAST.

### Genome Annotation

Transposable elements were identified with RepeatModeler (v2.02) and the reference was softmasked with RepeatMasker (v4.1.4) (Smit et al. 2015; Flynn et al. 2020). Six *H. arizonensis* RNA libraries were imported from NCBI (PRJNA340270) and used in the EASEL (v1.5) pipeline along with the softmasked draft genome and arthropoda OrthoDB (v11) protein sequences to predict protein-coding genes (Webster et al. n.d.). The invertebrate training set was configured to filter false-positive predictions. The same inputs were utilized in the GeneMark-ETP (Bruna et al. 2024) gene prediction tool, which is part of the BRAKER3 (v3.0.2) pipeline (Gabriel et al. 2024). EASEL (filtered) and GeneMark (hmm) genes were independently mapped to the Pore-C chromosome-level assembly with Liftoff (v1.6.3) (Shumate and Salzberg 2021). The resulting GFF files were combined using the AGAT (v1.2) toolkit (Dainat et al. 2023). Protein sequences were extracted and scanned for protein domains using Pfam-A.hmm (v3.1b2) and HMMER (v3.3.2) (Eddy 2011; Mistry et al. 2021). Sequences without a domain were removed. Final summary statistics, including BUSCO, were run on the filtered proteins. Finally, EnTAP (v1.0.1) was run with the complete RefSeq database (v208) at 70/70 coverage to functionally annotate the predicted proteins (Hart et al. 2020). Summary statistics, BUSCO, and EnTAP were also run on the longest isoforms to represent the unique gene space.

## Supplementary Material

evae097_Supplementary_Data

## Data Availability

The nanopore long reads used for the genome assembly have been deposited under the NCBI Bioproject PRJNA1072625. This references SAMN39896521 (standard nanopore), SAMN39747025 (ultralong nanopore) and SAMN41385265 (Pore-C). The assembly and annotation are hosted, with all project code on the following Gitlab: https://gitlab.com/PlantGenomicsLab/hadrurus-arizonensis-genome-assembly-and-annotation (DOI: 10.5281/zenodo.11086778).

## References

[evae097-B1] Open2C, Abdennur N, Fudenberg G, Flyamer IM, Galitsyna AA, Goloborodko A, Imakaev M, Venev SV. Pairtools: from sequencing data to chromosome contacts. bioRxiv:2023.02.13.528389. 10.1101/2023.02.13.528389, 2023, preprint: not peer reviewed. PMC11164360 38809952

[evae097-B2] Ballesteros JA, Santibáez-López CE, Baker CM, Benavides LR, Cunha TJ, Gainett G, Ontano AZ, Setton EVW, Arango CP, Gavish-Regev E, et al Comprehensive species sampling and sophisticated algorithmic approaches refute the monophyly of Arachnida. Mol Biol Evol. 2022:39(2):msac021. 10.1093/molbev/msac021. 35137183 PMC8845124

[evae097-B3] Bruna T, Lomsadze A, Borodovsky M. GeneMark-ETP: automatic gene finding in eukaryotic genomes in consistency with extrinsic data. bioRxiv:2023.01.13.524024. 10.1101/2023.01.13.524024, 2024, preprint: not peer reviewed.

[evae097-B4] Challis R, Kumar S, Sotero-Caio C, Brown M, Blaxter M. Genomes on a Tree (GoaT): a versatile, scalable search engine for genomic and sequencing project metadata across the eukaryotic tree of life. Wellcome Open Res. 2023:8:24. 10.12688/wellcomeopenres.18658.1. 36864925 PMC9971660

[evae097-B5] Cheng H, Concepcion GT, Feng X, Zhang H, Li H. Haplotype-resolved de novo assembly using phased assembly graphs with hifiasm. Nat Methods. 2021:18(2):170–175. 10.1038/s41592-020-01056-5. 33526886 PMC7961889

[evae097-B6] Coddington JA, Colwell RK. Arachnids. In: Levin SA, editor. Encyclopedia of biodiversity. 1st ed. San Diego (CA): Academic Press; 2001. p. 199–218.

[evae097-B7] Dainat J, Hereñú D, Murray KD, Davis E, Crouch K, Agostinho N, Zollman Z. NBISweden/AGAT: AGAT-v1.2.0. Zenodo. 2023. 10.5281/zenodo.8178877.

[evae097-B8] De Coster W, Rademakers R. NanoPack2: population-scale evaluation of long-read sequencing data. Bioinformatics. 2023:39(5):brad311. 10.1093/bioinformatics/btad311. PMC10196664 37171891

[evae097-B9] Durand NC, Robinson JT, Shamim MS, Machol I, Mesirov JP, Lander ES, Aiden EL. Juicebox provides a visualization system for Hi-C contact maps with unlimited zoom. Cell Syst. 2016:3(1):99–101. 10.1016/j.cels.2015.07.012. 27467250 PMC5596920

[evae097-B10] Durand NC, Shamim MS, Machol I, Rao SSP, Huntley MH, Lander ES, Aiden EL. Juicer provides a one-click system for analyzing loop-resolution Hi-C experiments. Cell Syst. 2016a:3(1):95–98. 10.1016/j.cels.2016.07.002. 27467249 PMC5846465

[evae097-B11] Eddy SR . Accelerated profile HMM searches. PLoS Comput Biol. 2011:7(10):e1002195. 10.1371/journal.pcbi.1002195. 22039361 PMC3197634

[evae097-B12] Flynn JM, Hubley R, Goubert C, Rosen J, Clark AG, Feschotte C, Smit AF. RepeatModeler2 for automated genomic discovery of transposable element families. Proc Natl Acad Sci USA. 2020:117(17):9451–9457. 10.1073/pnas.1921046117. 32300014 PMC7196820

[evae097-B13] Gabriel L, Brůna T, Hoff KJ, Ebel M, Lomsadze A, Borodovsky M, Stanke M. BRAKER3: Fully automated genome annotation using RNA-seq and protein evidence with GeneMark-ETP, AUGUSTUS and TSEBRA. bioRxiv:2023.06.10.544449. 10.1101/2023.06.10.544449, 2024, preprint: not peer reviewed. PMC11216308 38866550

[evae097-B14] Gao B, Wang Y, Diaby M, Zong W, Shen D, Wang S, Chen C, Wang X, Song C. Evolution of pogo, a separate superfamily of IS630-Tc1-mariner transposons, revealing recurrent domestication events in vertebrates. Mob DNA. 2020:11(1):25. 10.1186/s13100-020-00220-0. 32742312 PMC7386202

[evae097-B15] Graham MR, Jaeger JR, Prendini L, Riddle BR. Phylogeography of the Arizona hairy scorpion (*Hadrurus arizonensis*) supports a model of biotic assembly in the Mojave Desert and adds a new Pleistocene refugium. J Biogeogr. 2013:40(7):1298–1312. 10.1111/jbi.12079.

[evae097-B16] Hart AJ, Ginzburg S, Xu MS, Fisher CR, Rahmatpour N, Mitton JB, Paul R, Wegrzyn JL. EnTAP: bringing faster and smarter functional annotation to non-model eukaryotic transcriptomes. Mol Ecol Resour. 2020:20(2):591–604. 10.1111/1755-0998.13106. 31628884

[evae097-B17] Howard RJ, Edgecombe GD, Legg DA, Pisani D, Lozano-Fernandez J. Exploring the evolution and terrestrialization of scorpions (Arachnida: Scorpiones) with rocks and clocks. Organismal Diversity and Evolution. 2019:19(1):71–86. 10.1007/s13127-019-00390-7.

[evae097-B18] Ivancevic AM, Kortschak RD, Bertozzi T, Adelson DL. Horizontal transfer of BovB and L1 retrotransposons in eukaryotes. Genome Biol. 2018:19(1):85. 10.1186/s13059-018-1456-7. 29983116 PMC6036668

[evae097-B19] Kerkis I, de Brandão Prieto da Silva AR, Pompeia C, Tytgat J, de Sá Junior PL. Toxin bioportides: exploring toxin biological activity and multifunctionality. Cell Mol Life Sci. 2017:74(4):647–661. 10.1007/s00018-016-2343-6. 27554773 PMC11107510

[evae097-B20] Kim D, Song L, Breitwieser FP, Salzberg SL. Centrifuge: rapid and sensitive classification of metagenomic sequences. Genome Res. 2016:26(12):1721–1729. 10.1101/gr.210641.116. 27852649 PMC5131823

[evae097-B21] Kolmogorov M, Yuan J, Lin Y, Pevzner PA. Assembly of long, error-prone reads using repeat graphs. Nat Biotechnol. 2019:37(5):540–546. 10.1038/s41587-019-0072-8. 30936562

[evae097-B22] Kuntner M . The seven grand challenges in arachnid science. Frontiers in Arachnid Science. 2022:1:1082700. 10.3389/frchs.2022.1082700.

[evae097-B23] Li H . Minimap2: pairwise alignment for nucleotide sequences. Bioinformatics. 2018:34(18):3094–3100. 10.1093/bioinformatics/bty191. 29750242 PMC6137996

[evae097-B24] Liu B, Shi Y, Yuan J, Hu X, Zhang H, Li N, Li Z, Chen Y, Mu D, Fan W. Estimation of genomic characteristics by analyzing k-mer frequency in de novo genome projects. arXiv:1308.2012. https://doi.org/1308.2012, 2013, preprint: not peer reviewed.

[evae097-B25] Long M, Betrán E, Thornton K, Wang W. The origin of new genes: glimpses from the young and old. Nat Rev Genet. 2003:4(11):865–875. 10.1038/nrg1204. 14634634

[evae097-B26] Lozano-Fernandez J, Tanner AR, Giacomelli M, Carton R, Vinther J, Edgecombe GD, Pisani D. Increasing species sampling in chelicerate genomic-scale datasets provides support for monophyly of Acari and Arachnida. Nat Commun. 2019:10(1):2295. 10.1038/s41467-019-10244-7. 31127117 PMC6534568

[evae097-B27] Manni M, Berkeley MR, Seppey M, Zdobnov EM. BUSCO: assessing genomic data quality and beyond. Curr Protocols. 2021:1(12):e323. 10.1002/cpz1.323. 34936221

[evae097-B28] Marburger S, Alexandrou MA, Taggart JB, Creer S, Carvalho G, Oliveira C, Taylor MI. Whole genome duplication and transposable element proliferation drive genome expansion in Corydoradinae catfishes. Proc R Soc B: Biol Sci. 2018:285(1872):20172732. 10.1098/rspb.2017.2732. PMC5829208 29445022

[evae097-B29] *medaka: Sequence correction provided by ONT Research*. (n.d.). Github. Retrieved May 4, 2023, from https://github.com/nanoporetech/medaka

[evae097-B30] Mikheenko A, Prjibelski A, Saveliev V, Antipov D, Gurevich A. Versatile genome assembly evaluation with QUAST-LG. Bioinformatics. 2018:34(13):i142–i150. 10.1093/bioinformatics/bty266. 29949969 PMC6022658

[evae097-B31] Mistry J, Chuguransky S, Williams L, Qureshi M, Salazar GA, Sonnhammer ELL, Tosatto SCE, Paladin L, Raj S, Richardson LJ, et al Pfam: the protein families database in 2021. Nucleic Acids Res. 2021:49(D1):D412–D419. 10.1093/nar/gkaa913. 33125078 PMC7779014

[evae097-B32] Pagel Van Zee J, Geraci NS, Guerrero FD, Wikel SK, Stuart JJ, Nene VM, Hill CA. Tick genomics: the Ixodes genome project and beyond. Int J Parasitol. 2007:37(12):1297–1305. 10.1016/j.ijpara.2007.05.011. 17624352

[evae097-B33] Proctor HC, Smith IM, Cook DR, Smith BP. Chapter 25—Subphylum Chelicerata, class Arachnida. In: Thorp JH, Rogers DC, editors. Thorp and Covich's Freshwater Invertebrates. 4th ed. Volume I: Ecology and General Biology. Cambridge (MA): Academic Press; 2015. p. 599–660.

[evae097-B34] Rhie A, Walenz BP, Koren S, Phillippy AM. Merqury: reference-free quality, completeness, and phasing assessment for genome assemblies. Genome Biol. 2020:21(1):245. 10.1186/s13059-020-02134-9. 32928274 PMC7488777

[evae097-B35] Rittschof CC, Robinson GE. Chapter five—behavioral genetic toolkits: toward the evolutionary origins of complex phenotypes. In: Orgogozo V, editor. Current Topics in Developmental Biology. Vol. 119. Cambridge (MA): Academic Press; 2016. p. 157–204. 10.1016/bs.ctdb.2016.04.001 27282026

[evae097-B36] Roach MJ, Schmidt SA, Borneman AR. Purge haplotigs: allelic contig reassignment for third-gen diploid genome assemblies. BMC Bioinformatics. 2018:19(1):460. 10.1186/s12859-018-2485-7. 30497373 PMC6267036

[evae097-B37] Sanggaard KW, Bechsgaard JS, Fang X, Duan J, Dyrlund TF, Gupta V, Jiang X, Cheng L, Fan D, Feng Y, et al Spider genomes provide insight into composition and evolution of venom and silk. Nat Commun. 2014:5(1):3765. 10.1038/ncomms4765. 24801114 PMC4273655

[evae097-B38] Santibáñez-López CE, Aharon S, Ballesteros JA, Gainett G, Baker CM, González-Santillán E, Harvey MS, Hassan MK, Abu Almaaty AH, Aldeyarbi SM, et al Phylogenomics of scorpions reveal contemporaneous diversification of scorpion mammalian predators and mammal-active sodium channel toxins. Syst Biol. 2022:71(6)(6):1281–1289. 10.1093/sysbio/syac021. 35348798

[evae097-B39] Santibáñez-López CE, Ojanguren-Affilastro AA, Graham MR, Sharma PP. Congruence between ultraconserved element-based matrices and phylotranscriptomic datasets in the scorpion tree of life. Cladistics. 2023:39(6):533–547. 10.1111/cla.12551. 37401727

[evae097-B40] Santibáñez-López CE, Ojanguren-Affilastro AA, Sharma PP. Another one bites the dust: taxonomic sampling of a key genus in phylogenomic datasets reveals more non-monophyletic groups in traditional scorpion classification. Invertebr Syst. 2020:34(2):133–143. 10.1071/IS19033.

[evae097-B41] Schwager EE, Sharma PP, Clarke T, Leite DJ, Wierschin T, Pechmann M, Akiyama-Oda Y, Esposito L, Bechsgaard J, Bilde T, et al The house spider genome reveals an ancient whole-genome duplication during arachnid evolution. BMC Biol. 2017:15(1):62. 10.1186/s12915-017-0399-x. 28756775 PMC5535294

[evae097-B42] Shumate A, Salzberg SL. Liftoff: accurate mapping of gene annotations. Bioinformatics. 2021:37(12):1639–1643. 10.1093/bioinformatics/btaa1016. 33320174 PMC8289374

[evae097-B43] Smit AFA, Hubley R, Green P. RepeatMasker Open-4.0 2015. http://www.repeatmasker.org

[evae097-B44] Šťáhlavský F, Kovařík F, Stockmann M, Opatova V. Karyotype evolution and preliminary molecular assessment of genera in the family Scorpiopidae (Arachnida: Scorpiones). Zoology. 2021:144:125882. 10.1016/j.zool.2020.125882. 33278760

[evae097-B45] Thomas GWC, Dohmen E, Hughes DST, Murali SC, Poelchau M, Glastad K, Anstead CA, Ayoub NA, Batterham P, Bellair M, et al Gene content evolution in the arthropods. Genome Biol. 2020:21(1):15. 10.1186/s13059-019-1925-7. 31969194 PMC6977273

[evae097-B46] Wang Y, Jia L, Tian G, Dong Y, Zhang X, Zhou Z, Luo X, Li Y, Yao W. shinyCircos-v2.0: leveraging the creation of Circos plot with enhanced usability and advanced features. iMeta. 2023:2(2):e109. 10.1002/imt2.109. PMC10989951 38868422

[evae097-B47] Wang H, Liu B, Zhang Y, Jiang F, Ren Y, Yin L, Liu H, Wang S, Fan W. Estimation of genome size using k-mer frequencies from corrected long reads. arXiv:2003.11817. http://arxiv.org/abs/2003.11817, 2020, preprint: not peer reviewed.

[evae097-B48] Webster C, Fetter K, Zaman S, Vuruputoor V, Bhattarai A, Chinta V, Wegrzyn J. (n.d.). EASEL. GitLab. Retrieved 22 August 2023, from https://gitlab.com/PlantGenomicsLab/easel

[evae097-B49] wf-pore-c . (n.d.). Github. Retrieved March 13, 2024, from https://github.com/epi2me-labs/wf-pore-c

[evae097-B50] Wilson EB . The distribution of sperm-forming materials in scorpions. J Morphol. 1931:52(2):429–483. 10.1002/jmor.1050520205.

[evae097-B51] Zhou C, McCarthy SA, Durbin R. YaHS: yet another Hi-C scaffolding tool. Bioinformatics. 2023:39(1):btac808. 10.1093/bioinformatics/btac808. 36525368 PMC9848053

